# Discoveries Interview: Professor George A. Calin on non-coding RNAs

**DOI:** 10.15190/d.2014.24

**Published:** 2014-11-07

**Keywords:** Professor George A. Calin, interview

**Figure 1:**
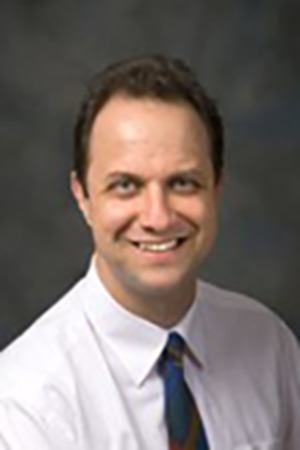
Professor George A. Calin

**Professor George A. Calin, MD, PhD,** received his MD and PhD degrees from “Carol Davila” University of Medicine and Pharmacy, Bucharest, Romania. Professor Calin continued his cancer genomics training in Doctor Massimo Negrini’s laboratory at University of Ferrara, in Italy. In 2000 he became a postdoctoral fellow at Kimmel Cancer Center in Philadelphia, PA, in Carlo Croce’s laboratory. He is presently a Professor in Experimental Therapeutics, Division of Cancer Medicine, and Co-Director of the Center for RNA Interference and Non-Coding RNAs, at MD Anderson Cancer Center, Houston, TX, USA.

Professor George Calin authored more than 259 scientific articles and received several awards and fellowships, including the 2013 Celgene Award for Clinical Research in Hematology, from Celgene.

**Together with Professor Carlo M. Croce and other collaborators, Prof. George Calin pioneered the idea that small non-coding RNAs – microRNA genes (miRNAs) are involved in human tumorigenesis^1^. He also proved that another family of ncRNAs, named ultraconserved genes (UCGs), are involved in human cancers and directly interact with miRNAs^2^. Professor Calin’s work is now focused on the translation of these discoveries to better diagnosis and treat cancer patients.

## 1. Can you define in simple words what are the non-coding RNAs and why are they important?

Non-coding RNAs and their short category of microRNAs are strangers in the genomic galaxy as they don’t codify for what was supposed to be the main product of any gene, a protein. In fact after the human genome was sequenced in early 2000’s, famous scientists were counting the number of coding genes and all without exception come with quite “empty hands” – instead of the 100K+ expected genes, the counts were barely reaching 25K. The rest of the genes were not yet accounted, as were not codifying for proteins, and they were located in between protein coding regions in the parts of the genomes considered “dark matter” by open minded scientists and simply trash cans of the genome by the “aficionados” of the dogma “one post-doc, five years of work, one protein”.

There are many categories of non-coding RNAs according to their lengths, and the shorter ones, the microRNAs, are about 20 nucleotides in length and are the most well studied at the present time. They are all regulators of protein levels and functions by multiple mechanisms, including complementarity binding to messenger RNAs as microRNAs work. Most of this mechanisms are not yet well understood and for sure there are many more to be identified.

The non-coding RNA genome field is the new Eldorado of molecular oncology for young and bright scientists as this new out-of-the box thinking is triggering fundamental discoveries. As microRNAs were recently found to be direct agonists of Toll-like receptors, a new avenue was open to explore their mechanisms of action and a to apply these findings to clinical practice by producing mutated miRNAs with higher or lower affinity to the receptor binding site.

What we have to keep in mind is that the majority of long non-coding RNAs have very short open reading frames of tens or a couple hundreds nucleotides, so still a lot of room for very short peptides to be produced, so next decade can be considered the time of micropeptides!! Certainly the last decade was the time of microRNAs in genomics and now we will try hard to translate this to clinical practice by using miRNAs as diagnostic biomarkers and in targeted therapy.

## 2. How our knowledge about them evolved over the time? What is your personal contribution in the field?

The knowledge in this field evolved from microRNAs as short degraded RNAs without any biological role to the main regulators of biological functions, as they are seen in present. Only this year over 10,000 papers will be published on a myriad of biological aspects and diseases in which miRNAs were found to play a role.

I was lucky enough to be at right time in the right place. While working with the world-renowned geneticist, Dr. Carlo Croce's at the Kimmel Cancer Center in Philadelphia, we discovered in 2002 the link between human cancers and microRNAs. Drs. DeVita and Rosenberg considered this discovery among the “major events in the cancer field” in their 2012 NEJM Anniversary review entitled “Two Hundred Years of Cancer Research”. Surely, my scientific life would not be so fulfilled without the contribution of my mentors, Dr Dragos Stefanescu in Romania, Dr Massimo Negrini in Italy and Dr Carlo Croce in US.

## 3. How our knowledge on ncRNAs will help understand and target human diseases? What will the field look like in 5-10 years?

I think the next 5 to 10 years will be fundamental for the non-codingRNA field. What both scientists and clinicians are looking for are successful clinical applications, developing new therapies using microRNAs as well as the identification of the potential role of miRNAs as biomarkers in early diagnosis or prediction of response to therapy. As tens of years of studies using protein coding genes produced few significant examples, the expectations are high on these new types of genes.

## 4. What advices do you have for young scientists?

I would recommend them simply to stay into science if they fill this is like a hobby for them, if they are coming smiling at the bench each day and they are able to adjust daily to new and unexpected responses of their experimental data. Also, I would suggest them to read at least one paper a day from as various fields of related science to their main focus; in this way they will be ready to understand what’s new on many topics and ready to apply this knowledge to their topic of interest. Also, I would advise them never give up in front of the colleague’s rumors and mistrust if they believe in their data, doesn’t matter how strange and against the current dogma looks like. I remember like it was yesterday, the year 2001 when my colleagues were telling me that I am working with degraded RNAs instead of true coding genes…Today, is my time to smile…

## 5. In your opinion, what are the most challenging, promising and/or the most rewarding areas of research? In your opinion, what are the most challenging, promising and/or the most rewarding areas of research?

Of course, I can simply answer the non-coding RNAs research… I think there is no proper answer to this question, as any area of research in which you are involved as a student, scientist, or clinician is the most rewarding for you as long as you will keep an open mind to new data and on how to better apply the new findings in the advantage of the patients.
